# Incidence of right heart failure and its impact on survival outcomes after implantable left ventricular assist device implantation

**DOI:** 10.1007/s00380-025-02651-2

**Published:** 2026-01-21

**Authors:** Hidetomi Takahashi, Masahiko Ando, Minoru Ono

**Affiliations:** Department of Cardiovascular Surgery, The University of Tokyo Hospital, Hongo 7-3-1, Bunkyo , Tokyo 113-8655 Japan

**Keywords:** Ventricular assist device, Right heart failure, Survival

## Abstract

In Japan, median waiting time of heart transplantation (HT) is estimated as more than 5 years. Internationally, they report relatively high incidence of right heart failure (RHF) during left ventricular assist device (LVAD) support. However, the analysis based on nationwide survey in Japan is still limited. Therefore, we aimed to clarify the predictor and incidence of RHF after LVAD implantation in contemporary cohort, and its impact on their survival outcomes. Adult patients who underwent LVAD implantation at our institution from 2007 to 2023 were retrospectively reviewed. Those eventually weaned off and those bridged from paracorporeal VAD were excluded, and finally 178 patients were enrolled. We evaluated the incidence of RHF, post-LVAD survival, and predictors of RHF or on-device mortality. Five-year on-device survival was 78.5%. The incidence of early RHF was 3.4% and its cumulative incidence at 5 year was 22.1%. While 91.5% of the cohort underwent LVAD as BTT, post-LVAD survival was not significantly worse with RHF (*p* = 0.107). Cox regression analyses demonstrated preoperative severe TR and dilated phase of hypertrophic cardiomyopathy or arrhythmogenic right ventricular cardiomyopathy were the independent predictors of RHF and on-device death (hazard ratio (HR) 8.59, 95% confidence interval (CI) 3.68–20.0, HR 2.62, 95% CI 1.08–6.34, respectively). The incidence of early RHF was 3.4% and its cumulative incidence at 5 year was 22.1%, which was relatively low. Although RHF was not significantly associated with worse 5-year survival after LVAD implantation, we would need a large, nation-wide study to further address this issue.

## Introduction

Right heart failure (RHF) after left ventricular assist device (LVAD) implantation has a serious impact on patients’ survival outcomes. Based on the STS INTERMACS registry, the rate of early RHF was recently reported as 24%, and late RHF (de novo RHF at 6 months or later) was persistent, easy to recur, and its 3-year survival was only 51% (while 73% in no-RHF group) [1]. Despite our decent and continuous efforts to elucidate this untoward issue, all the concerns associated with RHF, such as its prediction, prevention, etiology, and management [2–8], remain to be fully clarified to date. Especially in Japan, for serious donor shortage and HT allocation policy as of 2024, mean waiting time was 1877 days (5.1 years) among those who underwent HT in 2022 [9]. Moreover, nearly all HT candidates need to be on BTT-LVAD for years, even with marginal right ventricular (RV) function [10]. To address this issue, we are now building up Status IA allocation rule, so that the HT candidates with significantly tenuous RV function could get earlier HT. Fortunately, although we had been required to make a relatively high standard for HT candidacy, our RHF-free survival has been excellent so far, which was more than 80% at 5-years after LVAD implantation [11]. However, from now on, we must pay even more attention toward the risk and the actual effect of RHF on current LVAD outcomes, to reveal truly high-risk population in the waitlist and to create both efficient and fair allocation system. Therefore, in the present study, we aimed to clarify the predictor and incidence of RHF after LVAD implantation in contemporary cohort, and its impact on their survival outcomes.

## Materials and methods

### Study population

All patients with the age of > 15 years who underwent LVAD implantation at our institution from November 2007 to October 2023 were retrospectively reviewed. We excluded those bridged from a paracorporeal VAD (n = 43) and those eventually weaned off from an LVAD (n = 12), to avoid bias by preoperative optimization and relatively preserved ventricular functions, respectively. Eventually, 178 patients were enrolled in the main analysis. Based on the occurrence of RHF during follow up, they are divided into two groups: A) RHF_No group (n = 149) and B) RHF_Yes group (n = 29). Between these groups, patients’ baseline demographics, operative findings and post-operative outcomes were compared. Definition of RHF was based on J-MACS criteria, which was made based on INTERMACS criteria reported elsewhere [1]. Current J-MACS definition of RHF (version 1.2) is basically the same as STS-INTERMACS definition (version 2.3). In these definitions, RHF is defined as “symptoms and signs of persistent RV dysfunction [central venous pressure (CVP) > 18 mmHg with a cardiac index < 2.0 L/min/m2 in the absence of elevated left atrial/pulmonary capillary wedge pressure (greater than 18 mmHg), tamponade, ventricular arrhythmias or pneumothorax] requiring RVAD implantation; or requiring inhaled nitric oxide or inotropic therapy for a duration of more than 1 week at any time after LVAD implantation”. To build up J-MACS registry database, all the VAD institutions in Japan are now required to report VAD-associated adverse events based on J-MACS definition. Therefore, we used J-MACS criteria to define RHF in the present study. This study followed the STROBE guidelines, the study protocol complied with the Declaration of Helsinki, and it was approved by the Institutional Review Board at the University of Tokyo Hospital (number; 3031-(4)). An opt-out informed consent protocol was used. The consent procedure was reviewed and approved by the Institutional Review Board at the University of Tokyo Hospital. None of the participants opted out of the study.

### Statistical analysis

All analyses were performed using R v4.0.2 (R Foundation for Statistical Computing, Vienna, Austria) software. Continuous variables are presented as median with interquartile range and compared with the Mann–Whitney U test. Categorical data are presented as number and percentage of the total and compared using the Fisher’s exact test. Kaplan–Meier analysis was used to assess cumulative incidence of RHF and post-operative survival, and log-rank tests were conducted to evaluate statistical differences between the groups. To identify preoperative variables associated with a hazard of RHF and on-device mortality, univariable and multivariable Cox regression analyses were used. We included the following 6 predictors in the analysis; age, gender, estimated glomerular filtration rate (eGFR) < 60 ml/minutes/1.73m2, INTERMACS profile 1 or 2, preoperative severe tricuspid regurgitation (TR), and specific etiologies (dilated phase hypertrophic cardiomyopathy; dHCM, and arrhythmogenic right ventricular cardiomyopathy; ARVC). Potential predictors were identified with a stepwise method using the Akaike information criterion.

## Results

**Fig. 1:**
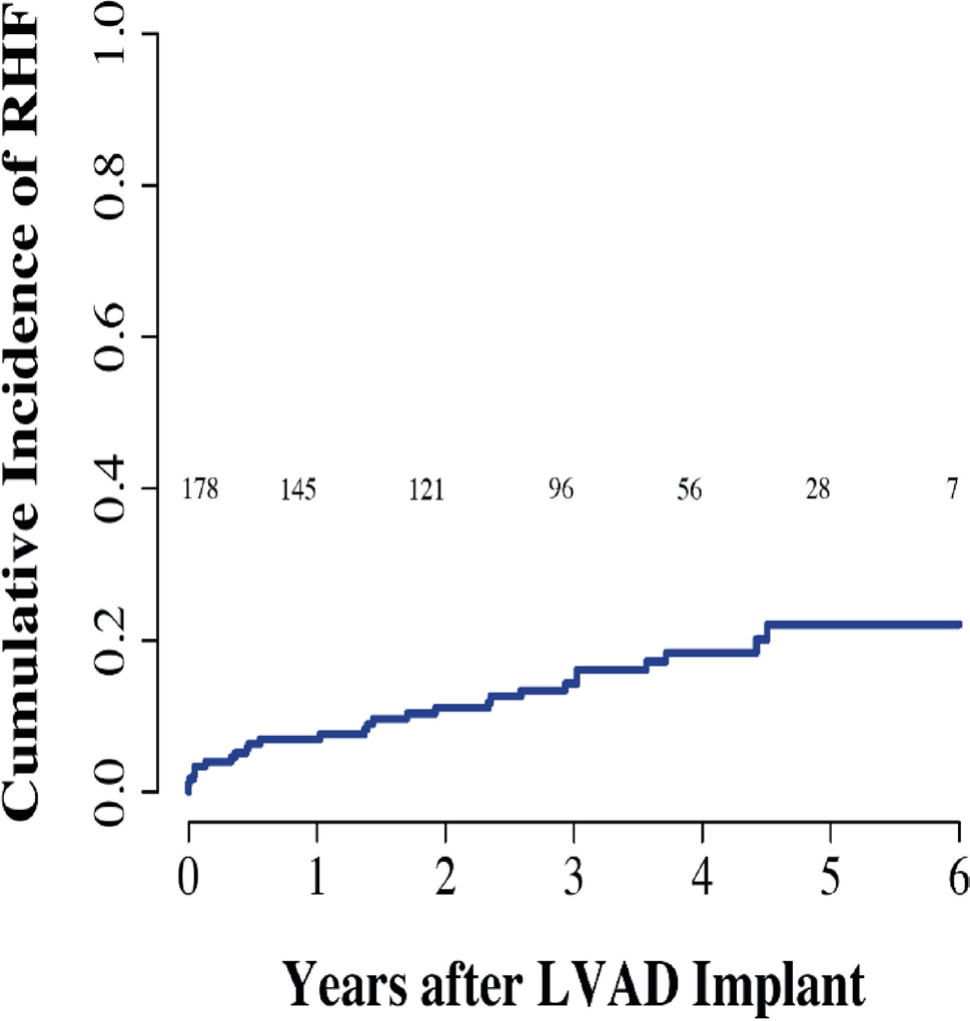
Cumulative incidence rate of right heart failure after LVAD implantation

Figure 1 demonstrates a cumulative incidence of RHF after LVAD implantation. Out of 178 patients included in the current analysis, RHF was seen in 29 patients (16.3%) during follow-up. Six of them occurred within one month after the implantation, indicating the incidence of early RHF (i.e. within 30 days after LVAD implantation) was 3.4%. Out of these 6 patients with early RHF, one patient required concomitant right ventricular assist device (RVAD) at the time of LVAD implantation, corresponding to early acute RHF in the definition by mechanical circulatory support academic research consortium [12]. Eventually the cumulative incidence of RHF at 5 years after LVAD implantation was 22.1%.

Table 1 demonstrates baseline profiles at LVAD implantation. There were no significant differences in overall baseline profiles. However, in primary disease, dilated phase of hypertrophic cardiomyopathy (dHCM) and arrhythmogenic right ventricular cardiomyopathy (ARVC) were more frequently observed in the RHF_Yes group (7.4 vs. 17.2%, 1.3 vs. 6.9%, respectively). The frequencies of non-ischemic dilated cardiomyopathy (DCM) were similar between the groups. Fifteen patients (8.4%) underwent LVAD implantation as destination therapy (DT) indication, and the rest of them was bridge-to-transplant (BTT) indication. In both groups, renal function was similar, but total bilirubin level was significantly higher in the RHF_Yes group (1.0 vs. 1.3 mg/dl, *p* = 0.028). Of note, the patients in the RHF_Yes group more frequently required preoperative ventilator support (2.0 vs. 13.8%, *p* = 0.014). No significant difference was observed in the J-MACS Risk scores (Table 1).

**Table 1:** Baseline profiles at LVAD implantation

	RHF_No (n = 149)	RHF_Yes (n = 29)	p value
Age	46 [36, 55]	44 [31, 53]	0.327
Female (%)	43 (28.9)	8 (27.6)	1.000
Height (cm)	169 [161, 173]	166 [160, 170]	0.278
Weight (kg)	56 [50, 63]	52 [49, 57]	0.073
Primary Disease (%)			0.142
DCM	100 (67.1)	20 (69.0)	
dHCM	11 (7.4)	5 (17.2)	
ICM	14 (9.4)	0 (0.0)	
ARVC	2 (1.3)	2 (6.9)	
Others	22 (14.8)	2 (6.9)	
Hypertension (%)	67 (45.0)	11 (37.9)	0.621
Diabetes (%)	34 (22.8)	5 (17.2)	0.675
Dyslipidemia (%)	42 (28.2)	3 (10.3)	0.074
COPD (%)	24 (16.1)	2 (6.9)	0.318
History of CVA (%)	19 (12.7)	2 (6.9)	0.647
DT (%)	14 (9.4)	1 (3.4)	0.490
Serum creatinine (mg/dl)	0.95 [0.77, 1.17]	1.03 [0.74, 1.32]	0.434
EGFR (ml/min/1.73m2)	63 [48, 86]	63 [46, 80]	0.595
Total bilirubin (mg/dl)	1.0 [0.6, 1.5]	1.3 [0.9, 1.7]	0.028
BNP (pg/ml)	553 [320, 1003]	763 [259, 949]	0.789
INTERMACS profile (%)			0.508
1	2 (1.3)	1 (3.4)	
2	61 (40.9)	16 (55.2)	
Preoperative support (%)			
Inotrope	101 (67.8)	21 (72.4)	0.140
IABP	46 (30.9)	12 (41.4)	0.375
Impella	7 (4.7)	0 (0.0)	0.504
VA-ECMO	2 (1.3)	1 (3.4)	0.986
Ventilator	3 (2.0)	4 (13.8)	0.014
J-MACS risk score	8.6 [7.0, 10.7]	9.4 [7.1, 12.6]	0.596

LVAD, left ventricular assist device; RHF, right heart failure; DCM, dilated cardiomyopathy; dHCM, dilated phase of hypertrophic cardiomyopathy; ICM, ischemic cardiomyopathy; ARVC, arrhythmogenic right ventricular cardiomyopathy; COPD, chronic obstructive pulmonary disease; CVA, cerebrovascular vascular accident; DT, destination therapy; EGFR, estimated glomerular filtration rate; BNP, brain natriuretic peptide; IABP, intra-aortic balloon pumping; VA-ECMO, veno-arterial extracorporeal membrane oxygenation; INTERMACS, Interagency Registry for Mechanically Assisted Circulatory Support; J-MACS, Japanese registry for Mechanically Assisted Circulatory Support

Table 2 shows cardiac functions at LVAD implantation. In the RHF_Yes group, LV dimensions were smaller (left ventricular end-diastolic dimension: 74 versus 61 mm, *p* = 0.005, left ventricular end-systolic dimension: 68 versus 57 mm, *p* = 0.004). Preoperative tricuspid regurgitation (TR) grade was more severe in the RHF_Yes group (severe TR: 4.0 vs. 27.6%, *p* < 0.001). There were no differences in the history of arrhythmia before the implantation.

**Table 2:** Cardiac functions at LVAD implantation

	RHF_No (n = 149)	RHF_Yes (n = 29)	p value
LVEF (%)	16 [12, 22]	18 [13, 25]	0.250
LVDd (mm)	74 [64, 80]	61 [55, 73]	0.005
LVDs (mm)	68 [57, 76]	57 [49, 68]	0.004
AR (%)			0.321
Moderate	2 (1.3)	1 (3.4)	
Severe	1 (0.7)	0 (0.0)	
MR (%)			0.447
Moderate	40 (26.8)	5 (17.2)	
Severe	21 (14.1)	2 (6.9)	
TR (%)			< 0.001
Moderate	19 (12.8)	1 (3.4)	
Severe	6 (4.0)	8 (27.6)	
History of Arrhythmia (%)			0.558
Af/AF	22 (14.8)	6 (20.7)	
VT/VF	14 (9.4)	4 (13.8)	

LVAD, left ventricular assist device; RHF, right heart failure; LVEF, left ventricular ejection fraction; LVDd, left ventricular end-diastolic diameter; LVDs, left ventricular end-systolic diameter; AR, aortic regurgitation; MR, mitral regurgitation; TR, tricuspid regurgitation; Af, atrial fibrillation; AF, atrial flutter; VT, ventricular tachycardia; VF, ventricular fibrillation

Table 3 demonstrates right ventricular function by echocardiography and right heart catheter data. No significant differences were observed in echo parameters, such as RV end-diastolic and end-systolic area, RV fractional area change, and tricuspid annular plane systolic excursion. Central venous pressure (CVP) and CVP/pulmonary capillary wedge pressure (PCWP) ratio were significantly higher in the RHF_Yes group (CVP: 6 vs. 10 mmHg, *p* = 0.001, CVP/PCWP ratio: 0.37 vs. 0.45, *p* = 0.009). There was no significant difference in the RV stroke work index between the groups.

**Table 3:** Right ventricular function and right heart catheter data

	RHF_No (n = 76)	RHF_Yes (n = 17)	*p* value
Echo parameters			
RV end-diastolic area (cm2)	25 [19, 33]	27 [22, 50]	0.152
RV end-systolic area (cm2)	19 [13, 27]	22 [16, 40]	0.198
RV fractional area change (%)	27 [20, 38]	28 [21, 33]	0.811
TAPSE (cm)	1.5 [1.3, 1.8]	1.4 [0.8, 1.8]	0.530
Right heart catheter parameters			
CVP (mmHg)	6 [4, 9]	10 [7, 16]	0.001
PCWP (mmHg)	18 [11, 26]	18 [14, 22]	0.858
CVP/PCWP Ratio	0.37 [0.25, 0.48]	0.45 [0.38, 1.11]	0.009
RV stroke work index	5.8 [4.4, 8.0]	6.5 [2.3, 8.2]	0.456
Systolic PAP (mmHg)	33 [24, 46]	32 [22, 54]	0.804
Diastolic PAP (mmHg)	18 [12, 25.50]	18 [14, 26]	0.758
Mean PAP (mmHg)	24 [17, 34]	23 [18, 37]	0.827
Cardiac index (L/min/m2)	1.89 [1.52, 2.35]	1.94 [1.60, 2.41]	0.404
PVR (wood unit)	2.25 [1.44, 3.50]	1.82 [1.19, 3.33]	0.201
Mixed venous O2 (%)	63.6 [56.1, 69.8]	62.3 [56.2, 72.0]	0.654

RHF, right heart failure; RV, right ventricular; TAPSE, tricuspid annular plane systolic excursion; CVP, central venous pressure; PCWP, pulmonary capillary wedge pressure; PAP, pulmonary artery pressure; PVR, pulmonary vascular resistance

Operative characteristics at LVAD implantation are shown in Table 4. No significant differences were found in redo (i.e. history of prior sternotomy) or LVAD device selections. Notably, we just started HeartMate 3 (Abbott, Illinois, U.S.A.) implantation in 2019, so HeartMate 3 rate was only 17.4% among the cohort, indicating the data includes prior generation devices, such as EVAHEART (Sun Medical Technology Research Corp, Nagano, Japan), DuraHeart (Terumo, Tokyo, Japan), Jarvik2000 (Jarvik Heart Inc, New York, U.S.A.), and HeartMate II (Abbott, Illinois, U.S.A.), which may provide worse outcomes as compared to a newer generation device. Tricuspid procedures were more frequently conducted in the RHF_Yes group, particularly tricuspid valve replacement (1.3 vs. 13.8%, *p* = 0.005).

**Table 4:** Operative characteristics at LVAD implantation

	RHF_No (n = 149)	RHF_Yes (n = 29)	*p* value
Redo (%)	27 (18.1)	2 (6.9)	0.221
First device (%)			0.234
HeartMate II	43 (28.9)	7 (24.1)	
HeartMate 3	29 (19.5)	2 (6.9)	
HVAD	19 (12.8)	1 (3.4)	
Jarvik 2000	19 (12.8)	8 (27.6)	
EVAHEART	28 (18.8)	8 (27.6)	
DuraHeart	11 (7.4)	3 (10.3)	
AV procedure (%)			0.384
AVR	13 (8.7)	1 (3.4)	
Park’s stitch	6 (4.0)	–	
MV annuloplasty (%)	38 (25.5)	8 (27.6)	0.934
TV procedure (%)			0.005
TVR	2 (1.3)	4 (13.8)	
Annuloplasty	60 (40.3)	13 (44.8)	

LVAD, left ventricular assist device; RHF, right heart failure; AV, aortic valve; AVR, aortic valve replacement; MV, mitral valve; TV, tricuspid valve; TVR, tricuspid valve replacement

In Table 5, early and late outcomes after LVAD implantation are shown. No significant difference was found in the in-hospital mortality (3.4 vs. 6.9%, *p* = 0.610). Duration of ICU stay, dialysis rate, and prolonged ventilator support rate (more than 72 h) were all worse in the RHF_Yes group. Notably, during mean follow-up of 5.4 years in the present study, our transplant rate was still around 50% (40.3 vs. 55.2%, *p* = 0.201), although more than 90% of the entire cohort underwent LVAD implantation as BTT indication (Table 1). On-device mortality tended to be higher in the RHF_Yes group (15.4 vs. 31.0%, *p* = 0.082). Among the RHF_Yes group, median time to RHF occurrence was 1.4 years after LVAD implantation. Out of 29 patients in the RHF_Yes group, two patients (6.9%) required RVAD implantation. One case was a fifties-year-old gentleman who underwent Jarvik 2000 implantation for DCM with LMNA gene mutation. Three years later, he underwent aortic valve plasty (Park’s stitch), complicated with concomitant RVAD implantation, and eventually deceased for multiple organ failure (MOF) at day 30. The other case was a forties-year-old lady who underwent HeartMate 3 implantation, tricuspid annuloplasty, and RVAD implantation for DCM with significant biventricular failure. She also died of MOF at day 25.

**Table 5:** Early and late outcomes after LVAD implantation

	RHF_No (n = 149)	RHF_Yes (n = 29)	*p* value
In-hospital mortality (%)	5 (3.4)	2 (6.9)	0.610
ICU stay (days)	6 [4, 7]	7 [5, 13]	0.024
PostOp maximum creatinine (mg/dl)	1.18 [0.90, 1.58]	1.38 [0.88, 1.68]	0.347
In-hospital outcomes (%)			
Dialysis	8 (5.4)	5 (17.2)	0.022
Stroke	11 (7.4)	3 (10.3)	0.301
Prolonged vent	9 (6.0)	6 (20.7)	0.033
Follow-up (years)	4.8 [2.7, 7.7]	5.9 [3.0, 8.5]	0.377
Device exchange (%)	24 (16.1)	3 (10.3)	0.611
Transplant (%)	60 (40.3)	16 (55.2)	0.201
Waiting time (years)	3.5 [2.0, 4.6]	3.2 [2.5, 4.8]	0.611
On-device mortality (%)	23 (15.4)	9 (31.0)	0.082
Time to RHF (years)	–	1.4 [0.3, 2.6]	–
RVAD (%)	–	2 (6.9)	–

LVAD, left ventricular assist device; RHF, right heart failure; ICU, intensive care unit; PostOp, postoperative; RVAD, right ventricular assist device

**Fig. 2:**
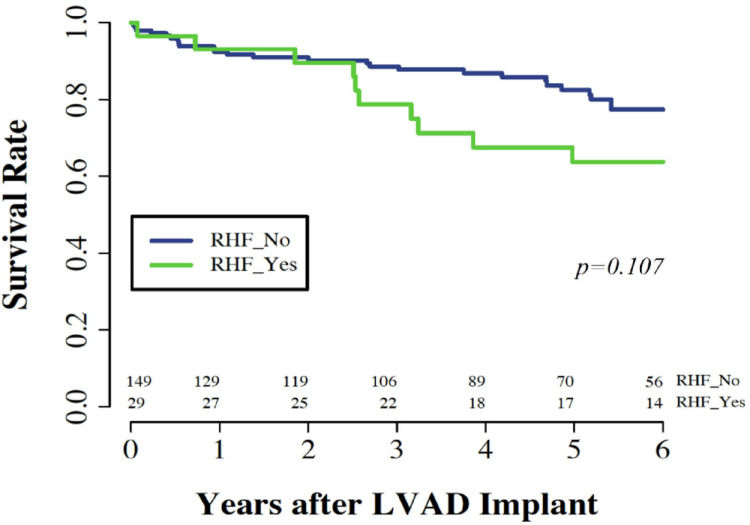
On-device survival rate after LVAD implantation. LVAD, left ventricular assist device

Figure 2 demonstrates on-device survival after LVAD implantation. A 5-year on-device survival was 78.5%, even with HeartMate 3 rate of 17.4% in the cohort, indicating most of the patients were implanted with prior generation devices (Table 4). Post-LVAD survival was not significantly worse in the RHF_Yes group (*p* = 0.107). Univariable and multivariable Cox regression analysis for the events of RHF and on-device death are shown in Table 6. In the model for RHF, preoperative severe TR and dHCM/ARVC etiology were the independent predictor of the event (preoperative severe TR: hazard ratio (HR) 8.59, 95% confidence interval (CI) 3.68–20.0, *p* < 0.001, dHCM/ARVC etiology: HR 2.62, 95% CI 1.08–6.34, *p* = 0.032). In the model for on-device mortality, preoperative severe TR was the independent predictor of the event (preoperative severe TR: HR 2.83, 95% CI 1.15–7.01, *p* = 0.024).

**Table 6:** Cox regression analysis for the events (RHF and On-device death)

Model for RHF	Univariable		Multivariable	
	HR (95% CI)	*p* value	HR (95% CI)	*p* value
Age	0.99 (0.97–1.02)	0.670	0.99 (0.96–1.02)	0.590
Female	1.01 (0.44–2.28)	0.987	–	–
EGFR < 60	0.69 (0.34–1.44)	0.329	–	–
INTERMACS Profile 1 or 2	1.97 (0.94–4.13)	0.072	–	–
Preoperative severe TR	7.99 (3.48–18.3)	< 0.001	8.59 (3.68–20.0)	< 0.001
dHCM and ARVC	2.64 (1.13–6.18)	0.026	2.62 (1.08–6.34)	0.032

Model for on-device death	Univariable		Multivariable	
	HR (95% CI)	*p* value	HR (95% CI)	*p* value
Age	1.04 (1.01–1.08)	0.007	1.03 (0.99–1.07)	0.074
Female	0.53 (0.20–1.37)	0.189	–	–
EGFR < 60	0.44 (0.22–0.91)	0.023	0.72 (0.31–1.67)	0.446
INTERMACS Profile 1 or 2	1.50 (0.74–3.03)	0.259	–	–
Preoperative severe TR	3.34 (1.36–8.15)	0.008	2.83 (1.15–7.01)	0.024
dHCM and ARVC	0.56 (0.13–2.37)	0.436	–	–

RHF, right heart failure; HR, hazard ratio; CI, confidence interval; EGFR, estimated glomerular filtration rate; INTERMACS, Interagency Registry for Mechanically Assisted Circulatory Support; TR, tricuspid regurgitation; dHCM, dilated phase of hypertrophic cardiomyopathy; ARVC, arrhythmogenic right ventricular cardiomyopathy

## Discussion

The main findings of the present study are summarized in the following four points. (1) 5-year on-device survival was 78.5% (Fig. 2), which was satisfactory, given that most of patients were implanted with prior-generation devices. (2) The incidence of early RHF was only 3.4% and its cumulative incidence at 5 years was 22.1% (Fig. 1), which is low as compared to the prior studies [1–3, 7]. 3) Although nearly all the patients in the present study underwent LVAD implantation as a BTT indication, HT rate remained approximately 50% at follow-up of 5–6 years, and the survival after LVAD was not significantly worse in the RHF_Yes group (*p* = 0.107). (4) Multivariable Cox regression analysis demonstrated that preoperative severe TR and the etiology of dHCM/ARVC were the independent predictors of the RHF (HR 8.59, 95% CI 3.68–20.0, *p* < 0.001, HR 2.62, 95% CI 1.08–6.34, *p* = 0.032, respectively), and preoperative severe TR was the independent predictors of on-device death (HR 2.83, 95% CI 1.15–7.01, *p* = 0.024). This low incidence of RHF after LVAD implantation would be worth reporting. Identification of the reason why such a low incidence of RHF was achievable in BTT population would provide some hints of how to stratify and prevent the risks of RHF, even in DT population.

RHF after LVAD implantation has a remarkable negative impact on patient survival outcomes, but to date, its etiology, prediction, prevention and management are not yet fully clarified, even in the US and Europe [1–8]. The recent publications on the incidence of RHF are summarized in Table 7. Based on the single center analysis in the United Kingdom, Gonzalez-Fernandez et al. reported that the incidence of early RHF was 46.9% and 29.9% among them required temporary RVAD (12.8% of the entire cohort), which was quite high [3]. While in the US, Kapelios et al. [1] and Rame et al. [7] reported that those were 24.0% and 26.0%, respectively, which were also high. In the present study, the incidence of early RHF was only 3.4%, based on J-MACS RHF definition, which is based on INTERMACS RHF definition. As for the incidence of late RHF, especially years after LVAD implantation, only limited data are available. This is because most of VAD patients would undergo HT in the US and Europe, otherwise they would decease within years. INTERMACS registry data demonstrated that the overall incidence of RHF was 37.8%, severe RHF was observed in 16.7% of the entire cohort. [2]. Based on EUROMACS registry, Nersesian et al. reported that the incidence of RHF at 3 year was 15 to 20% [5]. In the setting of these data, the present study showed that the cumulative incidence of RHF (including both early and late) at 5 year was 22.1% (Fig. 1), which is acceptable.

**Table 7:** Summary of prior publications on the risks and effects of RHF on LVAD outcomes

References	N, study period, population	Definition of RHF in the study	Summary of main findings
Gonzalez-Fernandez et al. [3]	N = 156 2009–2018 (HVAD) Single center, UK HVAD only	EUROMACS RHF Definition (summary) Early RHF: RVAD, continuous inotropic support for more than 14 days, or nitric oxide ventilation for more than 48 h after LVAD implantation Late RHF: Rehospitalization after more than 30 days of LVAD implantation	1. Incidence of early RHF was 46.9% and 29.9% among them required temporary RVAD (12.8% of the entire cohort) 2. Incidence of late RHF was 10.3%, with a median follow-up of 607 days (interquartile range 281 to 1065 days) 3. Significant preoperative TR was the strongest predictor of late RHF (hazard ratio 5.50, 95% confidence interval [1.34 to 22.58]; *p* = 0.02)
Rame et al. [7]	N = 6118 2014–2017 STS INTERMACS	STS INTERMACS RHF Definition (summary) RHF characterized by both of the following: Elevated CVP (> 16 mm Hg), dilated IVC by echo, or clinical findings of elevated jugular venous distension Presence of peripheral edema, ascites/palpable hepatomegaly, or worsening hepatic/renal dysfunction (total bilirubin > 2.0 mg/dL, creatinine > 2.0 mg/dL)	1. Prevalence of RHF at 1, 3, 6, and 12 months after LVAD implant was 26.0%, 10.2%, 9.1%, and 9.0%, respectively 2. For those with no RHF at 3 months, there was a low incidence of subsequent RHF at 6 and 12 months 3. The lack of RHF at 3 months, compared with mild and moderate RHF, was associated with a lower 12-month cumulative incidence of mortality (6.9% vs. 16.7% vs. 28.1%; *p* < 0.0001)
Kapelios et al. [1]	N = 5537 2014–2016 STS INTERMACS	STS INTERMACS RHF Definition	1. Prevalence of RHF at 1 month was 24% and RHF persisted at 12 months in 5.3% 2. Higher BUN, previous tricuspid valve surgery, severely depressed RV systolic function, and centrifugal LVAD were associated with RHF at 3 months 3. Patients with persistent RHF at 3 months had the lowest 2-year survival (57%) while patients with de novo RHF or RHF which resolved by 3 months had more favorable survival outcomes (75% and 78% at 2 years, respectively; *p* < 0.001)
Chamogeorgakis et al [2]	N = 6632 2013–2020 STS INTERMACS	STS INTERMACS RHF Definition	1. Incidence of RHF was 37.8%, and severe RHF was observed in 1105 patients (16.7% of the entire cohort) 2. Nearly all (97.7%) of severe RHF were the early one 3. Of 1079 patients with severe and early RHF, 769 were treated with inotropic support, 223 underwent temporary RVAD, and 77 received durable RVAD 4. Those received durable RVAD provided the worst outcomes
Nersesian et al. [5]	N = 834 2014–2023 EUROMACS	EUROMACS RHF Definition	1. Over-all incidence of RHF at 3 year was 15–20% 2. Those with a smaller LVEDD (< 65 mm) were older, more often female, and more ischemic cardiomyopathy 3. Smaller LVEDD group had a higher risk of postoperative RHF and worse 1-year survival
Molina et al. [4]	N = 1921 2016–2023 MOMENTUM3 HeartMate3 only	Symptoms and signs of persistent RV dysfunction requiring RVAD implantation, or requiring inhaled nitric oxide, or inotropic therapy for a duration of more than 1 week at any time after LVAD implantation	1. Those with a smaller LVEDD (< 55 mm) were older, more often female, and more ischemic cardiomyopathy 2. Compared with the LVEDD > 55 mm group, operative death was higher in the smaller LVEDD group (14.8 vs. 5.7%; *p* = 0.0007), and survival at 2 year was lower (63.3 vs. 81.8%; *p* = 0.0002) 3. Smaller LVEDD group had more deaths due to RHF, both early (0–30 days; 7.4 vs. 2.0%; *p* = 0.001) and late (> 30 days; 12.0 vs. 4.8%; *p* = 0.003)

RHF, right heart failure; UK, United Kingdom; LVAD, left ventricular assist device; RVAD, right ventricular assist device; TR, tricuspid regurgitation; STS, Society of Thoracic Surgeons; INTERMACS, Interagency Registry for Mechanically Assisted Circulatory Support; CVP, central venous pressure; IVC, inferior vena cava; BUN, blood urea nitrogen; RV, right ventricle; LVEDD, left ventricular end-diastolic diameter; EUROMACS, European Registry for Patients with Mechanical Circulatory Support

Undoubtedly, we should be careful about such a comparative assessment of RHF incidence between the different studies conducted in different settings, periods, and countries. Although, the reported rate of RHF is low in the present study, it does not necessarily mean our results are “superior” to those reported in the preceding publications. Nevertheless, there would be several reasons why RHF rate is relatively low in this analysis. Firstly, preoperative volume optimization by proper medication and, if necessary, insertion of some sort of mechanical circulatory support device, such as intra-aortic balloon pump and Impella, is critically useful. Secondly, intra-operative meticulous deairing should be thoroughly performed by continuous monitoring of transesophageal echocardiography. Just a tiny cluster of air into right coronary artery could lead to a devastating RHF requiring mechanical support such as Impella RP [13], particularly in a case with impaired RV function. Thirdly, reduction of tricuspid regurgitation is another key strategy. As shown in Table 2, only 19.1% of the entire cohort had preoperative TR moderate or greater, and 44.4% of the entire cohort underwent tricuspid valve replacement (TVR) or tricuspid annuloplasty. This is indicating we tend to actively intervene the tricuspid valve, if morphologically concerned, even if TR grade itself is mild or less. In fact, Kinoshita et al. reported tricuspid annular enlargement could be associated with TR progression, especially in patients with atrial fibrillation [14]. Although there are several recent reports that raise a question on the efficacy of concomitant tricuspid valve procedure at LVAD implantation [15, 16], their follow up periods are limited and the waiting time of HT is totally different from Japan. The intervention to aortic insufficiency (AI), is crucial to prevent RHF, as well as the intervention to tricuspid valve. Hatano et al. reported that the exacerbation of AI also leads to an increase in the afterload of the RV to induce late RHF, for the possible interrelationship between RHF, AI, and ventricular arrhythmia [17]. For this reason, we conducted concomitant aortic valve surgery in 11.2% of the entire cohort (Table 4), in the setting that the significant AR rate (moderate or severe) was only 2.2% (Table 2). Further domestic investigations are necessary to address these issues. Fourthly, both postoperative frequent speed adjustment based on the TTE findings and/or right heart catheterization results and appropriate medications to alleviate RV-PA decoupling might have worked. Particularly in the ICU, daily bedside TTE and right heart pressure measurement are essential. In an acute phase, we normally do not target high pump speed to prevent septal shift, and titrate the pump speed up slowly as needed. Our group previously reported the utility of right heart catheterization study with saline loading to evaluate the RV preload reserve function of patients with an LVAD [18]. If a low RV stroke work index change is observed with saline loading, we would consider an administration of nitric oxide while on a ventilator and, thereafter, oral sildenafil or other medications to improve possible RV-PA decoupling [19]. Recently, Ono et al. reported that the ratio of tricuspid annular plane systolic excursion (TAPSE) and PA systolic pressure could be a useful less-invasive indicator of RV-PA coupling [20], which would be beneficial in the post-LVAD managements.

There have been various preceding reports on the risk factors of RHF after LVAD implantation [17, 21–30]. Although in the present study, the number of RHF event was too low to conduct a decent multivariable analysis, preoperative severe TR was found to be an independent predictor of postoperative RHF and on-device mortality (Table 6), which is compatible to the prior publications. Although the reported risk factors of RHF might vary among these publication, most of them are reasonably associated with marginal RV function at baseline, such as severe TR, elevated RAP or RAP/PCWP ratio, reduced RV stroke work index, and reduced tricuspid annular plane systolic excursion (TAPSE), or other biological markers, such as elevated blood urea nitrogen (BUN), creatinine, and liver enzymes. However, the previous studies developed the center specific definitions of RHF without an external validation. Furthermore, some risk factors were established based on the data from prior-generation devices and may not be applicable to the current device. Schlöglhofer et al. conducted an analysis on HeartMate 3 and HVAD (Medtronic, Minnesota, U.S.A.), to demonstrate that RHF after LVAD was associated with HVAD, slower postoperative heart rate, and higher RAP [31]. Another recent report by Ruiz-Cano et al. on HeartMate 3 and HVAD cohorts showed that RAP/PCWP ratio > 0.55 and BUN > 44.5 mg/dL were the risk factors for RHF [32]. Finally, from the MOMENTUM-3 trial, Mehra et al. identified an IABP, DT indication, INTERMACS profile 1 or 2, and reduced glomerular filtration rate as predictors of RHF requiring a RVAD [33].

Another indispensable observation in the current study was the strong adverse effect of dHCM/ARVC etiology on the RHF events (Table 6). These non-DCM etiologies are associated with small LV cavity, that could have led to RHF. In fact, as shown in Table 2, the RHF_Yes group demonstrated small LV diastolic and systolic diameter (LVDd and LVDs), and our group previously demonstrated that small LV is an independent predictor of late-onset RHF after VAD implantation [24]. Small LV has been also included in the HeartMate 3 risk score model, which was established based on the MOMENTUM-3 trial [34].

## Study limitations

Our study has several limitations. First, it is necessarily limited by its retrospective, single-institution design, and as a single-center study, it reflects our own institutional treatment biases. The interpretation of the result should not be generalized to the nationwide and we would need a multi-centered study to address this issue. Secondly, to remove the effect of preoperative VAD on the outcome, and to make the study cohort more relevant to contemporary worldwide population, we excluded those on preoperative extracorporeal VAD. This might have led to the limitation of generalizability. Thirdly, the sample size for RHF_Yes group is small, rendering the study underpowered. RHF is multifactorial, however, multivariable analysis was quite difficult with such a low event rate, providing a concern about overfitting and statistical validity. For this reason, any identified predictors in the present study should be considered exploratory rather than conclusive. We would need more nationwide prospective analysis in the future. Fourthly, such a low event rate of RHF necessitated us to conduct multivariable regression analysis based on the combined exposures (i.e. the etiologies of dHCM and ARVC) and the combined outcomes (i.e. RHF and on-device death), although these combinations could be biologically plausible (both dHCM and ARVC tend to have small LV size, RHF should be associated with on-device death). And finally, the definition of RHF by J-MACS and INTERMACS criteria are not identical, and comparability with prior literature is another concern.

## Conclusions

Survival outcome and RHF incidence after LVAD implantation were satisfactory in the present study. The present study is also unique and worthwhile in that it clarified that preoperative severe TR and dilated phase of hypertrophic cardiomyopathy or arrhythmogenic right ventricular cardiomyopathy were the independent predictors of RHF and on-device death. However, since RHF was not significantly associated with worse 5-year survival after LVAD implantation and the number of RHF event is very limited in this cohort, we would need multi-center prospective analysis in the future, to further address the efficacy of RHF on the survival and to establish a more reasonable RHF-prediction model with such a low event rate.
